# Updated morphological description and molecular analysis of *Neoechinorhynchus* (*Neoechinorhynchus*) *iraqensis* (Acanthocephala: Neoechinorhynchidae) from *Planiliza abu* (Heckel) (Mugilidae) in Iraq

**DOI:** 10.2478/helm-2026-0004

**Published:** 2026-04-27

**Authors:** O. M. AMIN, A. CHAUDHARY, M. E. CARACCIOLO, A. H. ALI, N. Y. RUBTSOVA, M. H. AL-KURAIZY, H. S. SINGH, W. DE SOUZA

**Affiliations:** Institute of Parasitic Diseases, 11445 E. Via Linda, # 2-419, 85259 Scottsdale, Arizona; Molecular Taxonomy Laboratory, Department of Zoology, Chaudhary Charan Singh University, 250004 Uttar Pradesh, India; Centro Multiusuário para Análises de Fenômenos Biomédicos (CMABio), Universidade Estadual do Amazonas (UEA), Manaus Brasil; Department of Fisheries and Marine Resources, College of Agriculture, University of Basrah, Basrah, Iraq; Vice Chancellor, Glocal University, Delhi-Yamunotri Marg, Mirzapur Pole, Saharanpur, 247121, Uttar Pradesh, India; Laboratório de Ultraestrutura Celular Hertha Meyer, Instituto de Biofísica Carlos, Centro de Ciências da Saúde, Universidade Federal do Rio de Janeiro, 6 Chagas Filho, Rio de Janeiro, Brasil

**Keywords:** *Neoechinorhynchus iragensis*, *Planilizia abu*, Iraq, Revised morphology and molecular biology

## Abstract

The original description of *Neoechinorhynchus* (*N*.) *iraqensis* Amin, Al-Sady, Mhaisen, Bassat, 2001, from *Planiliza abu* (Heckel) in the Euphrates River, Iraq, is updated and completed, for the first time, using ocular microscopy and SEM, despite its repeated discovery by various observers in Iraqi waters. No descriptive accounts were provided in these earlier reports. Structures originally described in line drawings are currently demonstrated considerably more clearly in life-like 3-dimensional forms. New morphological features constituting partial revision are reported herein for the first time. Proboscis hooks, anterior trunk topography, reproductive structures organization, and egg anatomy were particularly emphasized. The DNA sequence of the small subunit of nuclear ribosomal RNA (*18S*) was used to substantiate its morphological distinction. Maximum likelihood and Bayesian inference analyses were performed, which showed that *Neoechinorhynchus* (N.) *iraqensis* formed a clade with strong bootstrap support and Bayesian posterior probability.

## Introduction

Since its original description from *Planiliza abu* (Heckel) in the Euphrates River near Al Faluja, in Iraq, *Neoechinorhynchus iraqensis* Amin, El-Sady, Mhaisen, Bassat, 2001, it has been reported from the same host in the Thi-Qar Province in southeastern Iraq by Mhaisen (2019) and from many species of fish from a number of geographical locations in Iraqi waters. Prior to its description, specimens of this parasite were erroneously identified as *Neoechinorhynchus agilis* (Rud., 1819), first by Habash and Daoud (1979) and later on in 28 reports from different parts of Iraq (Mhaisen, 2002), including that of Mhaisen *et al*. (1988) from *Liza abu* (= *Planiliza abu*) in Mehaijeran Creek. So far, *N. iraqensis*, as well as the erroneously reported *N. agilis*, have been reported by various authors in 21 fish host species from the southern Basrah region in different tributaries (Mhaisen *et al*,. 2014). Mhaisen and Abdul Ameer (2024) listed 22 different fish species that harbored *N. iraqensis* in Iraq from Tigris and Euphrates and Shatt Al-Arab Rivers, within Tigris River, Kurdistan Region Northeastern Iraq (Mhaisen & Abdullah, 2017), Nineveh Province, Kirkuk (Northern Iraq) and Baghdad Provinces mid Iraq (Mhaisen & Abdul Ameer, 2024), Salah Al-Din Province (Mhaisen *et al*., 2018), or from Shatt Al-Arab River off Basrah, southern Iraq (Mhaisen *et al*,. 2014), Euphrates River off Al-Anbar Province, western Iraq (Mhaisen *et al*,. 2017a), Euphrates River off Al-Najaf Al-Ashraf Province (Mhaisen & Al-Rubaie, 2016), Euphrates River off Al-Diwaniyah Province (Mhaisen *et al*., 2019), Euphrates River off Babylon Province (Mhaisen & Al-Rubaie, 2018), Thi Qar Province, southern Iraq (Mhaisen, 2019), or from southern marshes (Mhaisen *et al*., 2017b; Al-Musaedi & Alsaady, 2022)

Investigations of aspects of *N. iraqensis* other than field surveys included life cycle studies in the copepod intermediate host *Mesocyclops* (= *Cyclops*) *hyalinus* Rehberg, 1880 by Al-Sady (2009), and quantitative estimates of protein, carbohydrate, lipid, and nucleic acids by Hassan *et al*. (2016). This is the first complete report of the molecular analysis of *N. iraqensis* specimens.

## Materials and Methods

### Collections

We report 3 major collections of *N. iraqensis* specimens. (1) The original description and 5-line drawings (Amin *et al*., 2001) were based on weekly collections by Rana S. Al-Sady of many specimens of *N. iraqensis* from *Planiliza abu* (Heckel) (Mugilidae) between October 1998 and September 1999 in the Euphrates River at the town of Faluja (22°21'22"N; 43°46'58") in Anbar Province, west of Baghdad, Iraq. Acanthocephalans were collected from the intestines in 0.9 % saline solution or tap water, then fixed in 70 % ethanol after eversion of the proboscis. (2) In 2001, we received another collection of about 40 specimens of *N. iraqensis* collected by Dr. Fatima S. Al-Nasiri and Nada W. Hamoud from 20 of 224 examined individuals (8.92%) of *P.abu* from a fish farm near Baghdad. These specimens were processed for microscopy in the same manner as the original Al-Sady specimens were, as described below. (3) Most recently in 2024, we received 929 specimens collected from 274 of 884 examined individuals (30.99%) of *P abu*in the Al-Gharraf River (31°44 ‘60” to 31°58'74"N, 46°17'45" to 46°11'64"E), Thi Qar Province, southern Iraq between December 2022 and December 2023 (Table 1).

**Table 1.** Collections of *Neoechinorhynchus iraqensis* from Abu mullet *Planiliza abu* (Heckel) in Al-Gharraf River, Thi Qar Province, Southern Iraq. Extreme parameters are bolded.

No.	Months (Years)	Number examined fish	Number infected fish	Number of parasites	Percentage of infection	Intensity of infection
1	Dec 2022	23	-	-	-	-
2	Jan 2023	8	3	12	37.5	4.0
3	Feb 2023	16	2	4	12.5	2.0
4	Mar 2023	10	7	15	70.0*	**21.4**
5	Apr 2023	22	12	44	**54.5**	3.7
6	May 2023	103	76	464	**73.8**	6.1
7	Jun 2023	174	94	262	**54.0**	2.8
8	Jul 2023	94	39	69	41.5	1.8
9	Aug 2023	96	4	4	4.16	1.0
10	Sep 2023	90	13	13	14.1	1.0
11	Oct 2023	106	-	-	-	-
12	Nov 2023	71	17	30	23.9	1.8
13	Dec 2023	71	7	12	9.9	1.7
Total or mean		884	274	929	31.0	3.4

### Experimental protocol

Fish were collected periodically in the field using seine nets and then transferred on ice to the laboratory on the same day for processing. They were subsequently anesthetized before the intestinal tracts were removed for dissection, with a longitudinal slit, and parasite recovery. Acanthocephalans were placed in refrigerated water overnight or until the proboscides were everted, then transferred to cold 70 % ethanol for fixation and preservation.

### Management of specimens

Whole-mounted specimens from the first 2 collections were used for ocular microscopy. Specimens of the third collection were used for SEM and molecular analysis.

### Processing for microscopy

Worms from representative samples of the original and subsequent collections were stained in Mayer’s acid carmine, destained in ethanol and 40 % hydrochloric acid, dehydrated in ascending concentrations of ethanol, cleared in terpineol in 100 % ethanol, and whole-mounted in Canada Balsam. Specimens from more recent collections in 2023 were cleared in xylene. Type specimens were deposited in the US National Parasite Collection (Beltsville, Maryland) nos. 89469-89471.

### Optical microscopy

Images created for this presentation using optical microscopy were acquired using a Zeiss Axioskop Transmitted Nomarski DIC Phase Contrast Microscope Trinocular (Zeiss, Munich, Germany) and a Canon T3i EOS 600D DSLR Camera (Canon, Melville, New York). Images from the microscope were transferred from the laptop to a USB and stored for subsequent processing on a computer.

### SEM (Scanning electron microscopy)

Fixed parasite specimens in 70 % ethanol were prepared following established protocols (Lopes Torres *et al*., 2013), which included sequential dehydration through an ethanol gradient (70 % – absolute), critical point drying LEICA EM CPD 300 (Leica Microsystems, Wetzlar, Germany), and subsequent mounting on aluminum stubs using conductive double-sided carbon tape. Eggs were extracted from fixed adult females and adhered to glass coverslips using bovine skin gelatin (Sigma-Aldrich, St. Louis, Missouri), then processed using the same protocol applied to the parasite specimens. All samples were sputter-coated with a 20 nm-thick gold layer using a DII-29010SCTR Smart Coater (JEOL, Akishima, Japan). Observations were performed using a JEOL JSM-IT500HR SEM (JEOL). Images were taken at various magnifications. Samples were received under an acceleration voltage of 10 kV.

### Molecular methods

Two acanthocephalan specimens were processed for genomic DNA extraction using a DNeasy Blood and Tissue kit (Qiagen, Pleasanton, California)) according to the manufacturer’s instructions. The *18S* was amplified by polymerase chain reaction (PCR) using the primers Worm A, 1270R, and 930F, and Worm B (Littlewood and Olson 2001). PCR reactions (25 μl) consisted of 5 μl 1 mM deoxyribonucleotide triphosphates (dNTPs, Biotools, Spain), 0.80 μl of each primer, 2.5 μl of 10× Taq buffer (Biotools) with MgCl2, 0.70 μl of Taq polymerase (1 U; Biotools) and 12.20 μl of distilled water. PCR cycling comprised denaturation at 94 °C for 1 min, followed by 35 cycles of 94 °C for 1 min, annealing at 56 °C for 1 min, and extension at 72 °C for 1 min, followed by a terminal extension at 72 °C for 10 min. Sequencing reactions were electro-phoresed, and sequencing was performed using the BigDye Terminator v3.1 Cycle Sequencing Kit on an ABI 3130 Genetic Analyzer (Applied Biosystems, Foster City, California) according to the manufacturer’s protocol. Contigs were amassed and base-calling dissimilarities resolved using Codoncode Aligner version 3.5.4 (Codoncode Corporation, Dedham, Massachusetts). Sequences of the *18S* gene were deposited in the GenBank database.

Sequences obtained during the study of the *18S* gene were aligned using the Clustal W (Thompson *et al*,. 1994) algorithm implemented in MEGA 11 (Tamura *et al*., 2021) and manually adjusted. The aligned sequences were closely related to *Neoechinorhynchus* species retrieved from the GenBank database, as well as to sequences from other acanthocephalan species. Maximum likelihood (ML) and Bayesian Inference (BI) analyses were performed on the *18S* dataset. The ML tree was inferred using MEGA 11, whereas BI was inferred using TOPALi v2.5. The Model test program version 3.0 (Posada & Crandall, 2001) was used for inferring the best model of evolution for the *18S* data set. The best model of nucleotide substitution was estimated with the Akaike information criterion (AIC) as GTR + I + G. The ML analysis was run in MEGA 11 with 1,000 bootstrap replicates to support nodes. The genetic divergences among taxa were assessed in MEGA11 using the p-distance model. For BI analysis, the Markov chain Monte Carlo (MCMC) was run with 2 simultaneous runs of 4 chains over 1,000,000 generations, with every 100th tree saved, and with a “burn-in” set to the first 25 % of the trees. *Echinorhynchus gadi (*KF156880) was used as the outgroup. The *18S* sequences of *N. iraqensis* were deposited in the GenBank database (http://www.ncbi.nlm.nih.gov) (accession numbers: *18S*: PV776638, PV776640).

## Ethical Approval and/or Informed Consent

The authors declare that they have observed all applicable ethical standards.

## Results

The original morphological description by Amin *et al*. (2001) is enhanced by new perspectives now available through SEM images and ocular microscopy, as well as molecular analysis.

### New morphological features of N. iraqensis using SEM

Specimens of *N. iraqensis* were studied in detail and described with 5-line drawings by Amin *et al*. (2001) from the freshwater mullet *Planiliza abu* in the Euphrates River, Iraq. The shapes and measurements of all structures of the new materials reported in this paper, based on new collections from the Al-Gharraf River, Thi Qar Province, Southern Iraq, are similar to those reported in the original description. SEM revealed additional features not readily observed by optical microscopy, which are reported for the first time in Figures 1–16.

**Figs. 1–6.**
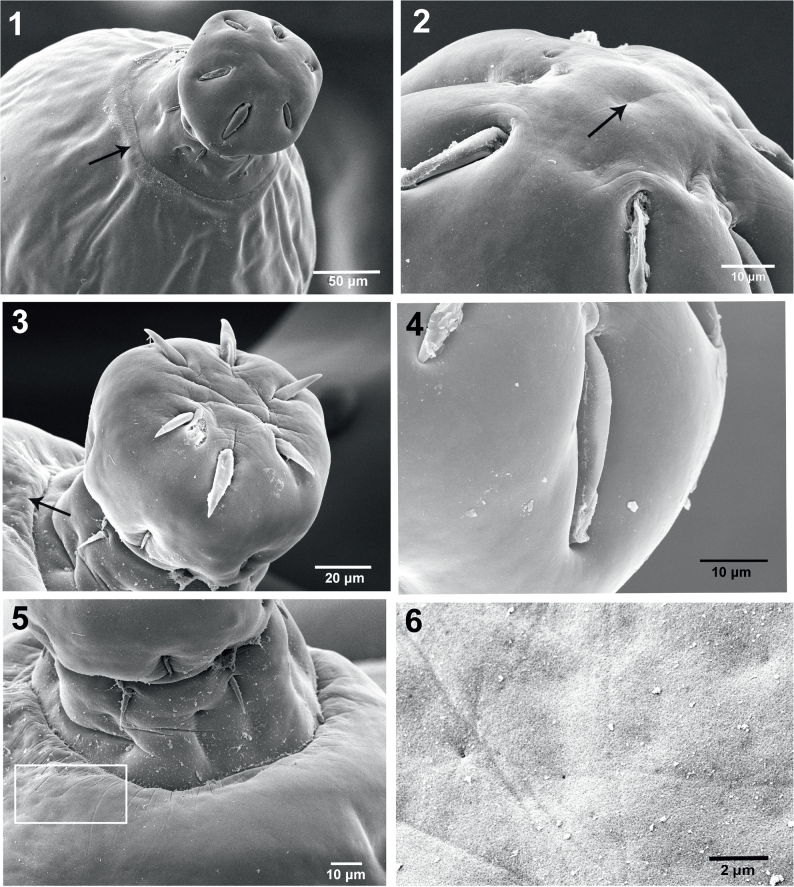
SEM images of specimens of *Neoechinorhynchus iraqensis* collected from *Planiliza abu* in the Al-Gharraf River, Thi Qar Province, southern Iraq. Fig. 1. A near apical perspective of the proboscis with deeply imbedded apical hooks, two sensory pores on the neck and a prominent cuticular thickening forming the girdle (arrow). Fig. 2. The apical end of a proboscis showing the indentation marking the apical organ. Fig. 3. A proboscis showing various sizes of apical hooks in 2 circles and part of the girdle (arrow). Fig. 4. An example of an anterior hook deeply embedded in the proboscis. Fig. 5. The posterior part of a proboscis and neck with sensory pores and an inset magnifying part of the girdle. Fig. 6. Inset from Figure 5 showing pores and radiating stellate indentations emanating from this cuticular layer of the girdle as well as prevalent micropores.

Figure 1 demonstrates the bulbous shape of the anterior proboscis with partially deeply embedded anterior hooks in 2 circles, two adjacent sensory pores among the few in the neck (arrow) and the characteristic cuticular thickening forming the girdle (black arrow).

**Figs. 7–12.**
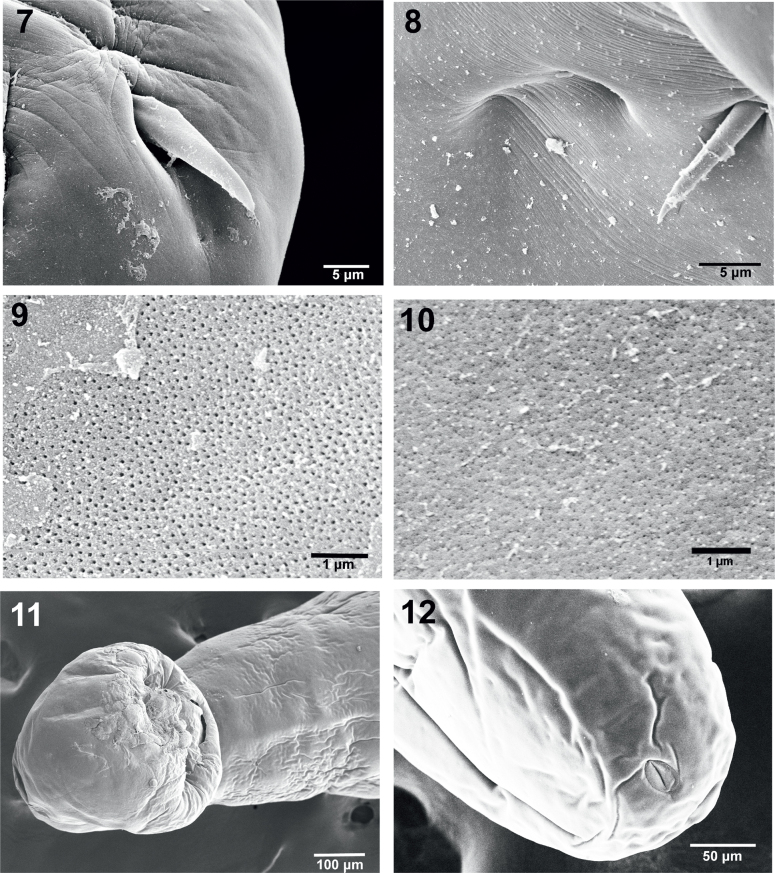
SEM images of specimens of *Neoechinorhynchus iraqensis* collected from *Planiliza abu* in the Al-Gharraf River, Thi Qar Province, southern Iraq. Fig. 7. A partially embedded posterior hook of one female. Fig. 8. A close up of two adjacent micropores just posterior to the posterior hook within the proboscis-neck continuous cuticular contour lines. Fig. 9. Micropores from the proboscis. Fig. 10. Micropores from the posterior trunk. Compare the two sets of micropores for size and distribution. Fig. 11. A ventral perspective of the bursa. Fig. 12. The posterior end of a female showing the sub-ventral position of the gonopore.

**Figs. 13–16.**
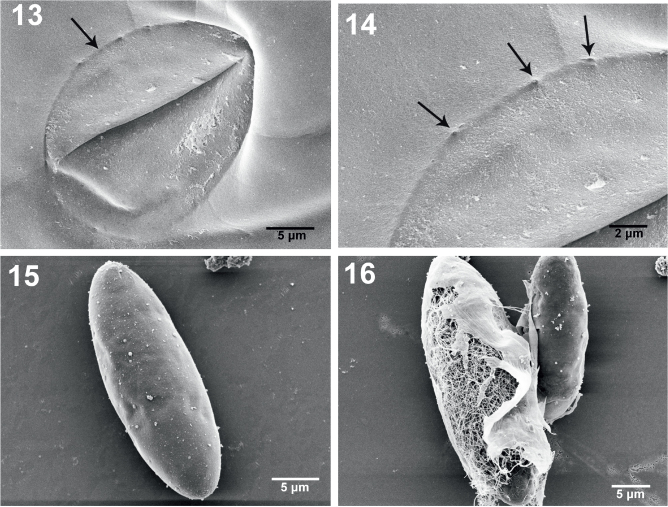
SEM images of specimens of *Neoechinorhynchus iraqensis* collected from *Planiliza abu* in the Al-Gharraf River, Thi Qar Province, southern Iraq. Fig. 13. A high magnification of a female gonopore showing the thick lips and a peripheral ovoid indentation (arrow). Fig. 14. A higher magnification of part of the ovoid indentation around the gonopore in Figure 13 showing sensory pores (arrows). Fig. 15. A ripe egg. Fig. 16. A ripe egg teased to show an extensive network of fibrils.

The apical end of the proboscis shows a depression marking the position of the apical organ. Figure 2 is a high magnification of the apical end of a proboscis showing the indentation marking the apical organ (arrow). Figure 3 provides an additional perspective on the proboscis anterior hooks, shown in 2 sizes and in different circles. The apical center of the proboscis also denotes the depression of the apical organ. The girdle partially appears to the far left (arrow). An anterior hook deeply recessed in the proboscis (Fig. 4) is a common occurrence. The posterior proboscis, sensory pore, neck and part of the girdle are partially magnified (inset) to show unique pores at the centers of stellate radiating depressions (Figs. 5, 6). The pits (Fig. 6) may be sensory in nature. Posterior hooks are also often deeply recessed in the proboscis (Fig. 7). Adjacent double sensory pores on the neck just posterior to the posterior hooks (Fig. 8) are often seen. Micropores were observed on all anatomical structures, including the proboscis (Fig. 9) and posterior trunk (Fig. 10), as well as on the girdle (Fig. 6). Note differences in size and distribution of pores in relation to the metabolic absorption of nutrients through the body wall. Figure 11 shows the shape and surface topography of the bursa; note the absence of apparent sensory structures in this perspective. The subterminal bi-fold female gonopore orifice (lips) appears at the posterior end of that specimen (Fig. 12). A high magnification of the female gonopore in Fig. 12 shows a marginal ovoid cuticular indentation (Fig. 13). Figure 14 shows a high magnification of the indentation surrounding the female gonopore in Figure 13 depicting characteristic pores (arrows) that may be sensory in nature. The egg of *N. iraqensis* is fusiform-ellipsoid with a smooth surface (Fig. 15). Still, it has a dense inner network of fibril layers (Fig. 16) that would eventually be freed after elimination for flotation and accessibility to the definitive host.

Taha *et al*. (2018) used poor specimens of *N. iraqensis* to produce the only other published record of SEM micrographs with inadequate interpretation.

**Figs. 17–22.**
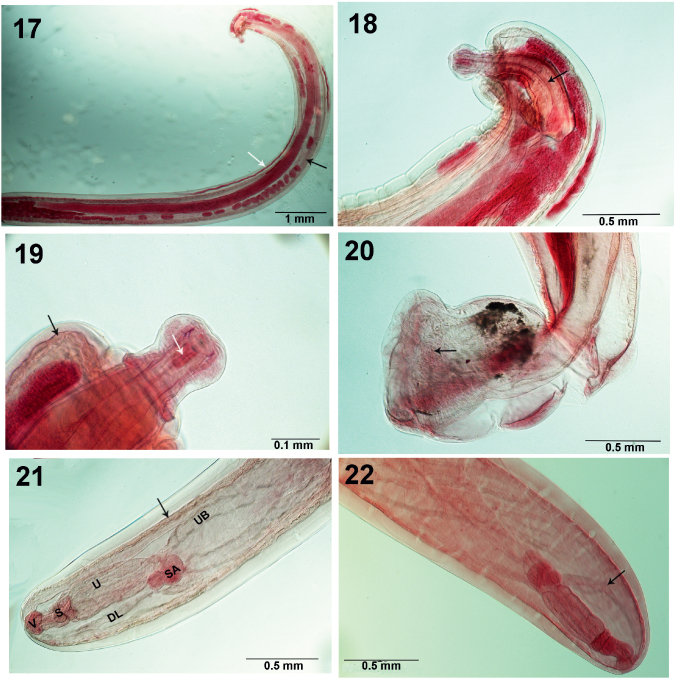
Light microscope images of specimens of *Neoechinorhynchus iraqensis* collected from *Planiliza abu* in the Al-Gharraf River, Thi Qar Province, southern Iraq. Fig. 17. Anterior part of a female specimen showing the proportional sizes of the proboscis, giant hypodermal nuclei (white arrow), and lemnisci (black arrow to smaller lemniscus). Fig. 18. A higher magnification of the anterior end of a female specimen showing the proboscis, curved receptacle, triangulate cephalic ganglion and apical sensory cord (arrow). Fig. 19. The proboscis, apical organ (white arrow) and beady body wall lining (black arrow) in 1 female specimen. Fig. 20. The bursa with possible sensory cells (arrow). Fig. 21. A female reproductive system showing vagina (V), sphincter (S), uterus (U), selective apparatus (SA), uterine bell (UB) primary dorsal bundle of ligaments (DL) (below) and the beady inner lining of the body wall (arrow). Fig. 22. Another perspective of a female reproductive system emphasizing the bundles of ligaments. The primary dorsal bundle is marked with an arrow.

New anatomical features of *N. iraqensis* using optical microscopy thorough optical microscopical observations of the new specimens from Al-Gharraf River also revealed many features that have not been previously reported and are herein described for the first time. Figures 17–22 supplement the original description by Amin *et al*. (2001) and overcome the limitations of the 5-line drawings used there.

The anterior trunk of a female specimen in the ovarian ball stage (Fig. 17) shows the small size of the proboscis compared to the trunk, the extensive size of some of the giant hypodermal nuclei (white arrow), and the size of the small lemniscus (black arrow) compared to the larger lemniscus. A higher magnification of the anterior trunk (Fig. 18) shows the shape of the anterior bulbous proboscis, the routinely curved proboscis receptacle with single wall and triangulate cephalic ganglion connected to the apical organ with prominent apical sensory cord (arrow). A higher magnification of the proboscis (Fig. 19) shows the extension of the apical organ through the anterior bulbous proboscis (white arrow) and anterior trunk wall with the characteristic beady inner lining (black arrow) found underlying the whole trunk wall. Detail of the bursa (Fig. 20) shows its bell shape, its connection to the posterior trunk, and what appear to be cellular structures at the bell constriction (arrow), suggestive of sensory elements. A detail of the female reproductive system (Fig. 21) demonstrates the angular vagina (V), strong sphincter (S), short and robust uterus (U), prominent selective apparatus (SA) with few cells, and the large, thick-walled uterine bell (UB). The primary dorsal bundle of ligaments (DL) (below) and the beady inner lining of the body wall (arrow), they are also prominent. Fig. 22 provides another perspective on the female reproductive system, emphasizing the various bundles of ligaments, starting with the primary dorsal bundle (arrow).

### Molecular data

**Fig. 23.**
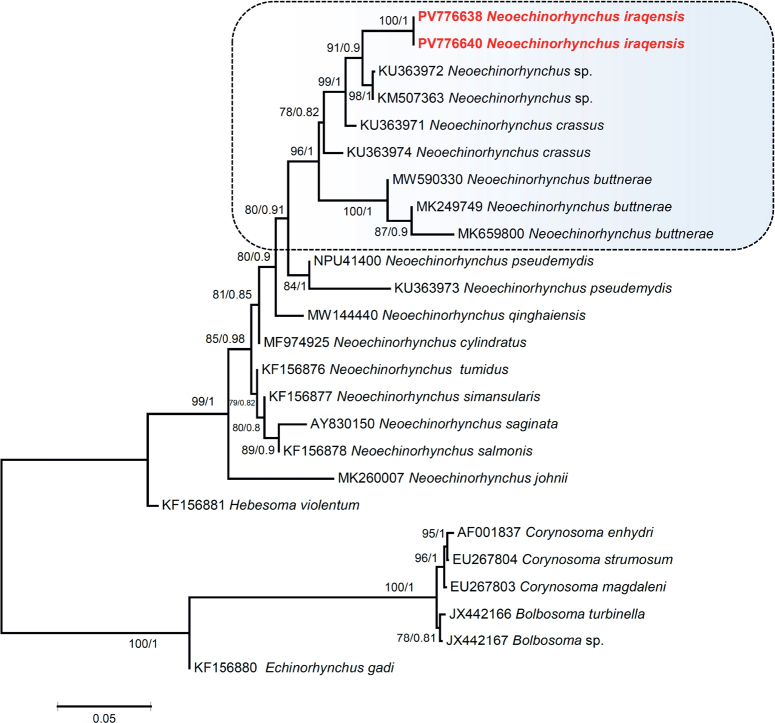
Phylogenetic trees obtained of the *18S* rDNA sequences of acanthocephalan species. Values on nodes are shown as maximum likelihood/Bayesian inference, the species names are shown next to the GenBank accession numbers, and the scale bars represent substitutions per site. Species sequenced in the present study are shown in bold red.

The phylogenetic tree of *18S* using ML and BI methods topologies were found to be similar that showed that representatives of *Neoechinorhynchus* formed a monophyletic clade (Fig. 23). The phylogenetic tree was well supported (BS = 99; PP = 1.00) and suggests *Neoechinorhynchus* a monophyletic origin, established along with *Hebesoma* (subgenus) as a sister group (Fig. 23). *Neoechinorhynchus iraqensis* was found close to two unknown *Neoechinorhynchus* species, KU363972 and KM507363 from Iran and China respectively with well supported values (BS = 91; BI = 1.00) (Fig. 23). The details about the related species present in the *N. iraqensis* clade are mentioned in Table 2. The subclade of *N. iraqensis* also comprised *Neoechinorhynchus crassus* Van Cleave, 1919, from Iran, and *Neoechinorhynchus buttnerae* from Brazil, both with well-supported values. *Neoechinorhynchus iraqensis* shows genetic divergence within its clade of 4 species, ranging from 1.75 to 2.61 ^¢^%, whereas the genetic divergence between *Neoechinorhynchus* sp. (KU363972), *Neoechinorhynchus* sp. (KM507363) and *N. iraqensis* is 1.75 %. Although Malyarchuk *et al*. (2014) provisionally renamed *N. crassus* specimens to a new species, because their *18S* rRNA gene sequences diverged extensively from North American specimens *of N. crassus*. Besides this, a study by Ali and Abdullah (2024) mentioned the molecular identification of *N. iraqensis* from *Planiliza abu* in Darbandikhan Lake, Iraq. However, the *18S* sequence from their study is still not registered in NCBI. No accession number is available in the paper (Ali & Abdullah 2024), that indicates the question on the amplified sequence. Therefore, we are unable to add their sequence to our analysis and cannot authentically state that the present study sequence is the first registered *18S* sequence for the species *N. iraqensis* from Iraq. Amin (2002) reported that a group with notably unequal lemnisci comprises 13 species, whereas 6 species have a thickened dorsal body wall, as reported by Amin and Heckmann (2009). Instead, the second set of species defined by Amin (2002) was established as a subgenus *Hebesoma*. Our analyses of the *18S* gene phylogenetic position show that *Hebesoma violentum* (=*Neoechinorhynchus* (*Hebesoma*) *violentus* Van Cleave, 1928) is close to *Neoechinorhynchus* species, and between *Hebesoma* and *Neoechinorhynchus*, a sister relationship is exhibited. Consequently, supplementary genomic markers are essential to delineate *Hebesoma*’s phylogenetic position in future studies. The genus *Neoechinorhynchus*, with a greater number of species, needs further study to determine its relationships within the genus and to provide a respected position in the trees.

**Table 2.** *Neoechinorhynchus* species information used for the phylogenetic analysis based on the *18S* gene sequences for species that are present in the clade of *Neoechinorhynchus iraqensis*. The species sequenced during this study is shown in bold.

Species	Host	Host origin	GenBank accession nos.	References
*Neoechinorhynchus* sp. GL-2015	*Capoeta aculeata*	Iran	KU363972	M. Adel and M. Dadar 2016 (Unpubl. data)
*Neoechinorhynchus* sp. XL-2014	Data not available	China	KM507363	X. Liu *et al*., 2014 (Unpubl. data)
*Neoechinorhynchus crassus*	*Capoeta aculeata*	Iran	KU363971 KU363974	M. Adel and M. Dadar, 2016 (Unpubl. data)
***Neoechinorhynchus iraqensis***	*Planiliza abu*	Iraq	PV776638, PV776640	Present study

## Discussion

In the present study, *N. iraqensis* was identified based on an integrated approach, using morphology and molecular data. The present study advances our knowledge of the diversity of *Neoechinorhynchus* species in West Asia, highlighting the requirement for keen studies on this group of parasites. Despite many reports on fish parasites from various Arabian Gulf waterways (e.g., Mhaisen, 2002), including a few descriptions, none has been as thoroughly studied as *N. iraqensis*. Nevertheless, our study of a large collection of new specimens demonstrated that the comprehensive original description of *N. iraqensis* by Amin *et al*. (2001) did not cover all facets of its morphology and anatomy. This presentation completes its morphological description using ocular microscopy and SEM images for the first time, with special emphasis on proboscis hooks, the anterior trunk landscape, the topography of reproductive structures, and egg anatomy. The new morphological and anatomical features added to the description of *N. iraqensis*, based on this new material as well as the original description, constitute a revision of the species description. Some of these features are unique to *N. iraqensis*, such as the beady inner lining of the body wall, and others are found in other species reviewed by Amin (2002) but not in this combination. Our study also adds a molecular confirmation of its identity using the *18S* gene. Molecular analyses of new acanthocephalan taxa, especially of the genus *Neoechinorhynchus* Stiles and Hassall, 1905, have not been used in the Arabian Gulf region in the past. We hope that we are introducing a new trend in taxonomic research on the acanthocephalans of fishes in this important region of the Middle East.

## Conflict of Interest

The authors declare no conflicts of interest.
